# Using Ecological Momentary Assessment to Redefine Postdialysis Fatigue in Patients with Kidney Failure

**DOI:** 10.1681/ASN.0000000650

**Published:** 2025-02-25

**Authors:** Cramer J. Kallem, Alaa A. Alghwiri, Jonathan Yabes, Sarah Erickson, Zhuoheng Han, Maria-Eleni Roumelioti, Jennifer L. Steel, Mark Unruh, Manisha Jhamb

**Affiliations:** 1 Renal-Electrolyte Division, Department of Medicine, University of Pittsburgh School of Medicine, Pittsburgh, Pennsylvania; 2 Division of General Internal Medicine, Center for Research on Heath Care Data Center, Department of Medicine and Biostatistics, University of Pittsburgh, Pittsburgh, Pennsylvania; 3 Department of Psychology, University of New Mexico, Albuquerque, New Mexico; 4 Division of Nephrology, Department of Internal Medicine, University of New Mexico School of Medicine, Albuquerque, New Mexico; 5 Department of Surgery, University of Pittsburgh School of Medicine, Pittsburgh, Pennsylvania; 6 Department of Psychiatry, University of Pittsburgh School of Medicine, Pittsburgh, Pennsylvania; 7 Department of Psychology, University of Pittsburgh School of Medicine, Pittsburgh, Pennsylvania

**Keywords:** chronic hemodialysis, quality of life

## Abstract

**Key Points:**

- Many patients with kidney failure on thrice-weekly in-center hemodialysis reported worsening of both fatigue and mood symptoms posthemodialysis.
- Retiring the term “postdialysis fatigue” and replacing it with “postdialysis syndrome” would more accurately describe this phenomenon.

**Background:**

Patients with kidney failure have a high symptom burden, and many report an acute exacerbation of symptoms immediately after in-center hemodialysis. Very few studies have used ecological momentary assessment to examine posthemodialysis patient-centered outcomes.

**Methods:**

Participants in the Technology-Assisted Collaborative Care trial completed an automated telephone-administered Daytime Insomnia Symptom Scale at four time points daily for 7 consecutive days. The Daytime Insomnia Symptom Scale yields four symptom domain scores: Positive Mood, Negative Mood, Alert Cognition, and Sleepiness/Fatigue. Posthemodialysis symptom domains and item-level scores were compared with similar time points on nonhemodialysis days using linear mixed models analyses. Mixed models were also used to explore the association of postdialysis symptom burden with demographic, psychosocial, hemodialysis treatment, and disease-specific characteristics. All analyses were adjusted for age, race, sex, and Charlson Comorbidity Index.

**Results:**

One hundred and fifty-six hemodialysis patients with available ecological momentary assessment data were evaluated (mean age=58±14 years, 55% men, 51% White). In the posthemodialysis period, patients reported significantly lower Positive Mood (mean difference [MD]=−0.22; 95% confidence interval [CI], −0.29 to −0.14) and Alert Cognition (MD=−0.13; 95% CI, −0.18 to −0.08) and higher Negative Mood (MD=0.12; 95% CI, 0.05 to 0.19) and Sleepiness/Fatigue (MD=0.51; 95% CI, 0.42 to 0.61) compared with nonhemodialysis days. The mean postdialysis symptom exacerbation total score was 0.70±1.21, representing an increase of 6% of the maximum scale range. In the postdialysis period, relative to nondialysis days, the mean decrease in Positive Mood and Alert Cognition scores equaled 4% and 2% of the maximum scale range, respectively; the mean increase in Negative Mood and Sleepiness/Fatigue scores equaled 2% and 9% of the maximum scale range, respectively.

**Conclusions:**

Patients with kidney failure reported worsening of both fatigue and mood symptoms posthemodialysis.

**Clinical Trial registry name and registration number:**

Technology Assisted Stepped Collaborative Care Intervention, NCT03440853.

## Visual Abstract

**Figure:**
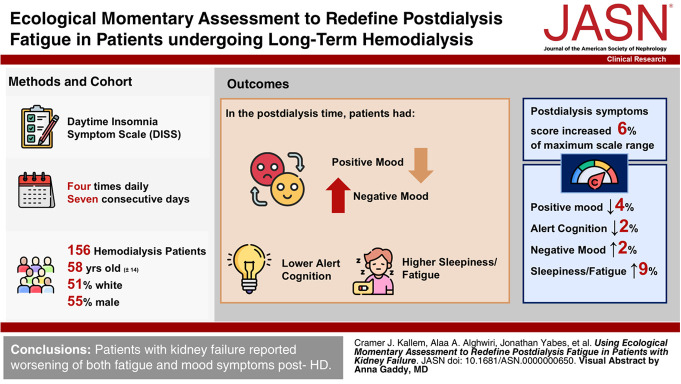

## Introduction

Patients with kidney failure have a high burden of physical and mood symptoms, which negatively affect their daily functioning and quality of life.^1–3^ In fact, most hemodialysis patients experience multiple concurrent symptoms, with fatigue, pain, depression, and anxiety being among the most common.^1,4–6^ Although these symptoms are common, they remain frequently underrecognized and undertreated in this population.^7,8^ Managing symptoms in patients with kidney failure may be particularly challenging as they are often unpredictable regarding onset, severity, and duration, which is also a significant contributor to patients' symptom distress.^1,6,9–11^

Postdialysis fatigue is perhaps the most well-studied example of the symptoms studied prehemodialysis and posthemodialysis.^12^ Although no universally accepted definition or characterization of postdialysis fatigue has been established, it is generally used to describe debilitating symptoms of fatigue (*e.g*., tiredness and physical exhaustion), which occur after dialysis.^13–18^ It is estimated that over 60% of hemodialysis patients are affected by postdialysis fatigue^12^; however, there is considerable heterogeneity in the intensity and duration of postdialysis fatigue symptoms.^19^

A number of poor health outcomes have been associated with postdialysis fatigue, including greater time to recover after dialysis,^20,21^ interdialytic weight gain,^22^ postdialysis hypertension,^22^ challenges completing activities of daily living,^20,21,23,24^ high-cost health care utilization,^25^ and mortality.^25^ Although the biologic mechanisms underlying postdialysis fatigue are not fully understood, several potential contributing factors have been implicated, including older age,^20–22^ greater dialysis vintage,^19,20^ comorbidities,^22^ anemia,^22^ depressive symptoms, ^17,25–28^ psychosocial stress,^29^ sedentary behavior,^26^ and elevations in proinflammatory cytokines^30^ and mixed findings regarding the influence of dialysis modality and dialysis prescription on postdialysis fatigue (see ref. 13 for a review).

Recently, the National Institute of Diabetes and Digestive and Kidney Diseases hosted a scientific workshop on postdialysis fatigue to describe the current evidence base and establish priority areas for future research.^31^ One of the themes that emerged from the conference proceedings was the lack of a formal case definition for postdialysis fatigue as well as critiques of the term “postdialysis fatigue.”^18,31^ Because patients often experience a range of debilitating physical, cognitive, and mood symptoms in the postdialysis period,^32,33^ use of the word “fatigue” may fail to adequately describe, and potentially minimizes, patients' postdialysis symptom experiences.^18,31^ Another salient theme was the need to develop assessment tools and strategies that can be used to measure postdialysis fatigue.^18,31^ Of note, it was recommended that these measures should include multiple domains (*e.g*., physical, cognitive, and mood symptoms) to better capture the multidimensional nature of postdialysis symptoms.^18,31^

Ecological momentary assessment (EMA), which involves repeated sampling of patient-reported symptoms in real time, is an especially promising approach to learning more about postdialysis fatigue and related symptoms.^34^ The use of repeated, frequent, measurements would allow for a closer examination of the temporal relationships between hemodialysis treatments and changes in symptom severity. Unlike retrospective self-report measures (*i.e*., the most common approach to measuring postdialysis fatigue),^12,13^ EMA helps to reduce recall bias, which is a particularly relevant concern given the cognitive symptoms many hemodialysis patients experience.^31,34,35^ However, very few studies have used EMA to examine hemodialysis patient-reported symptoms, and none have focused on symptom experiences in the postdialysis period.^10,36,37^

The aims of this study were to (*1*) describe patient-reported physical, cognitive, and mood symptoms in the postdialysis period; (*2*) compare symptom severity after dialysis versus the same period on nondialysis days; and (*3*) explore associations of postdialysis symptom exacerbation with sociodemographic, disease-specific, and psychosocial variables using EMA in a large sample of hemodialysis patients.

## Methods

### Setting and Participants

This was a secondary analysis of data collected as part of the Technology-Assisted stepped Collaborative Care trial,^38^ a randomized controlled trial to test the effectiveness of a collaborative care intervention on patient-reported symptoms in patients with kidney failure. Patients were enrolled between March 1, 2019, and December 31, 2021, from dialysis clinics in New Mexico and Pennsylvania. All patients were adults, receiving in-center thrice-weekly maintenance hemodialysis for at least 3 months, who screened positive for clinically significant levels of pain, fatigue, or depression and consented to receive treatment for these symptoms. All patients had conventional in-center hemodialysis prescriptions (*i.e*., three weekly sessions, each lasting 3–5 hours with standard blood flow rates [≥300 ml/min] and dialysate flow rates [≥500 ml/min]). The study was approved by the Institutional Review Boards at the University of New Mexico and University of Pittsburgh. See refs. 38 and 39 for a detailed description of the study procedures.

### Data Collection

All data analyzed in this study were collected before randomization. Sociodemographic and disease-specific data were extracted from patients' electronic medical records. Baseline questionnaires were administered by trained interviewers using computer-assisted telephone interviewing. EMA data were gathered using an automated telephone-administered version of the Daytime Insomnia Symptom Scale (DISS; described below), which patients completed at four daily time points (morning, early afternoon, late afternoon, and evening) for 7 consecutive days.

### Measures

#### EMA of Symptoms

The DISS is a 19-item measure of physical and mood symptoms.^40^ Items consist of a short question (*e.g*., “How _____ do you feel right now?”), each presenting a different physical, cognitive, or mood state (*e.g*., sleepy, clear-headed, happy, and stressed); patients then respond using a Likert scale (score range=1–7) with higher scores indicating greater endorsement of the physical, cognitive, or mood state. Principle components analysis has revealed four symptom domains for the DISS: Positive Mood, Negative Mood, Alert Cognition, and Sleepiness/Fatigue.^40^ Symptom domain scores range from 1 to 7 with higher scores indicating greater levels of the domain.

#### Baseline Measures of Sample Characteristics

See Supplemental Table 1 for descriptions of all measures of sample characteristics that were collected at baseline. Sociodemographic characteristics included age, sex, race, ethnicity, and a geographical location–based measure of social determinants of health–related burden. Disease-specific and treatment-specific characteristics included medication use, laboratory values, comorbidity burden, dialysis vintage, duration, adequacy, weekly schedule, and shift time. Psychosocial characteristics included measures of fatigue, sleep quality, pain, depressive symptoms, anxiety, physical activity, and social support.

### Data Analysis

DISS symptom domain scores were computed on the basis of the factor structure described by Buysse *et al.*^40^ for all patients at all time points. To compare patients' postdialysis symptom burden to their nondialysis day symptom burden, two variables were computed for all DISS items and symptom domains: *Postdialysis scores* were calculated by averaging patients' symptom domain and item-level scores for all time points after they completed dialysis on all dialysis days, and *nondialysis scores* were calculated by averaging patients' symptom domain and item-level scores for the same time points on all nondialysis days. Mixed modeling was then used to compare patients' postdialysis scores with their nondialysis scores. Sensitivity analyses were conducted by excluding second consecutive nondialysis day (example excluding Sunday for patients on Monday-Wednesday-Friday dialysis schedule).

To explore associations between sample characteristics and postdialysis symptom exacerbation, the following variables were computed for all patients: *Total postdialysis symptom burden scores* were calculated by adding patients' postdialysis Negative Mood and Sleepiness/Fatigue scores, and *total nondialysis symptom burden scores* were calculated by adding patients' nondialysis Negative Mood and Sleepiness/Fatigue scores. *Postdialysis symptom exacerbation scores* were then calculated by subtracting patients' total nondialysis symptom burden scores from their total postdialysis symptom burden scores. Higher postdialysis symptom exacerbation scores indicate greater symptom exacerbation in the postdialysis period. To help interpret the potential clinical significance of our findings, we calculated the proportion of mean differences (MDs) in symptom domain scores (postdialysis minus nondialysis) to the maximum scale range (range=6). In addition, given the diurnal variation in fatigue/sleepiness,^10^ we calculated the average increase in Sleepiness/Fatigue scores from wake-up time to bedtime (mean score at time point 4 minus mean score at time point 1). We used this as a clinically meaningful anchor to then calculate the proportion of MDs in Sleepiness/Fatigue scores (postdialysis minus nondialysis). Since there is no clear evidence that mood or cognition change in any universally predictable manner from morning to evening, we did not do similar analysis for other domains.

All primary analyses controlled for age, race, sex, and Charlson comorbidity index (CCI) scores. We also performed sensitivity analyses controlling for fatigue at baseline. Mixed modeling was used to explore the association of postdialysis symptom exacerbation scores with sociodemographic, disease-specific, and psychosocial sample characteristics. All analyses were performed using R version 4.3.3 (The R Foundation for Statistical Computing; 2024).

## Results

### Sample Characteristics

One hundred and fifty-six patients with EMA data were included in this study (mean age=58±14 years). Most were men (*n*=86, 55%) and White (*n*=79, 51%). See Table 1 for sample characteristics and baseline EMA symptom domain scores. Patient characteristics by dialysis shift were mostly similar (Supplemental Table 2). Although the morning shift patients reported lower levels of fatigue, anxiety, and depressive symptoms as compared with those in afternoon/evening shifts, there were no significant differences in the EMA domain scores.

**Table 1:** Baseline characteristics of participants who completed ecological momentary assessment at baseline in Technology-Assisted Collaborative Care study

Characteristic	Category or Statistic	Sample (*N*=156)
**Sociodemographic**		
Age	Mean years±SD	58±14
Sex, *n* (%)	Female	70 (45)
	Male	86 (55)
Race, *n* (%)	American Indian	21 (13)
	Black	46 (29)
	Other (>1 race/unknown)	10 (6)
	White	79 (51)
Ethnicity, *n* (%)	Hispanic	26 (17)
	Non-Hispanic	130 (83)
Social Deprivation Index	Mean±SD	55.3 (31)
**Disease-specific**		
Charlson Comorbidity Index	Mean±SD	4.8±1.8
Dialysis vintage	Mean years±SD	4±4
Hemoglobin	Mean g/dl±SD	11.2±1.3
Albumin	Mean g/dl±SD	4.0±0.4
Opioid medication use	Yes, *n* (%)	46 (30)
Antidepressant medication use	Yes, *n* (%)	57 (37)
Antihypertensive medication use	Yes, *n* (%)	131 (85)
*β* blocker medication use	Yes, *n* (%)	105 (68)
**Hemodialysis treatment**		
Dialysis schedule, *n* (%)	Monday-Wednesday-Friday	100 (64)
	Tuesday-Thursday-Saturday	56 (36)
Dialysis shift time, *n* (%)	Morning	75 (48)
	Midday and evening	81 (52)
Dialysis duration	Mean hours±SD	4.1±0.5
Dialysis adequacy, kt/v	Mean±SD	1.6±0.4
**Psychosocial**		
Functional Assessment of Chronic Illness Therapy–Fatigue	Mean±SD	28.3±11.0
Pittsburgh Sleep Quality Index	Mean±SD	8.9±3.4
Brief Pain Inventory-short form	Mean±SD	3.6±3.3
Beck Depression Inventory II	Mean±SD	15.3±8.4
General Anxiety Disorder-7	Mean±SD	5.9±4.8
Physical Activity Scale for the Elderly	Mean±SD	229.1±54.4
Multidimensional Scale of Perceived Social Support	Mean±SD	62.8±14.0
**EMA**		
Nondialysis symptom domain scores, mean±SD	Positive Mood	4.3±1.3
	Negative Mood	2.5±1.4
	Alert Cognition	4.9±1.0
	Sleepiness/Fatigue	4.1±1.7
Postdialysis symptom domain scores, mean±SD	Positive Mood	4.5±1.3
	Negative Mood	2.4±1.4
	Alert Cognition	5.0±0.9
	Sleepiness/Fatigue	3.6±1.7

Postdialysis=mean symptom domain scores for all time points after dialysis, on dialysis days; nondialysis=mean symptom domain scores for the same time points on nondialysis days. For Positive Mood and Alert Cognition, lower scores are worse; for Negative Mood and Sleepiness/Fatigue, higher scores are worse. EMA, ecological momentary assessment.

### Postdialysis Symptom Domains

**Figure 1:**
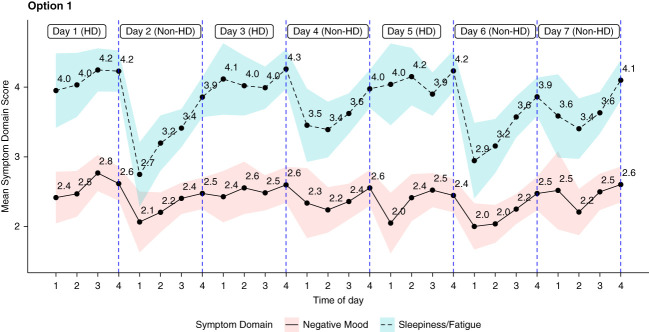
**Mean postdialysis and nondialysis Negative Mood and Sleepiness/Fatigue scores at all time points.** For Negative Mood and Sleepiness/Fatigue, higher scores are worse. Means plotted in Figure 1 include all EMA data that patients provided after their hemodialysis sessions on hemodialysis days as well as all EMA data that patients provided at the same time points on nonhemodialysis days. Error bands depict the 95% CI around the estimated mean at each time point. Time of day 1=morning, 2=early afternoon, 3=late afternoon, and 4=evening. For patients on a Monday-Wednesday-Friday dialysis schedule, day 1=Monday; for patients on a Tuesday-Thursday-Saturday dialysis schedule, day 1=Tuesday. Average Negative Mood scores by time point: 1=2.42, 2=2.52, 3=2.53, and 4=2.55. Average Sleepiness/Fatigue scores by time point: 1=3.60, 2=3.78, 3=3.80, and 4=4.09. CI, confidence interval; EMA, ecological momentary assessment; HD, hemodialysis.

**Figure 2:**
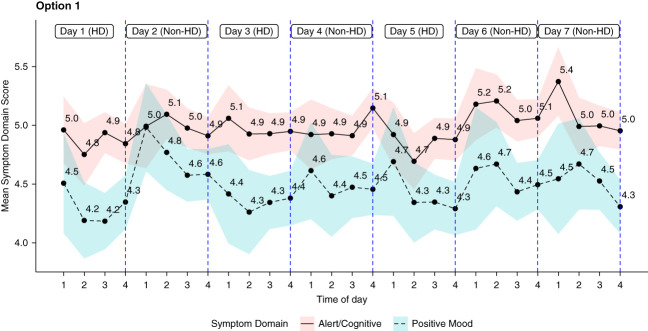
**Mean postdialysis and nondialysis Positive Mood and Alert Cognition scores at all time points.** For Positive Mood and Alert Cognition, higher scores are better. Means plotted in Figure 2 include all EMA data that patients provided after their hemodialysis sessions on hemodialysis days as well as all EMA data that patients provided at the same time points on nonhemodialysis days. Error bands depict the 95% CI around the estimated mean at each time point. Time of day 1=morning, 2=early afternoon, 3=late afternoon, and 4=evening. For patients on a Monday-Wednesday-Friday dialysis schedule, day 1=Monday; for patients on a Tuesday-Thursday-Saturday dialysis schedule, day 1=Tuesday. Average Alert Cognition scores by time point: 1=5.01, 2=4.92, 3=4.95, and 4=4.96. Average Positive Mood scores by time point: 1=4.47, 2=4.38, 3=4.39, and 4=4.40.

**Figure 3:**
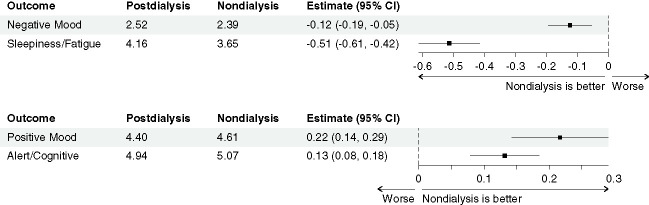
**Mean adjusted difference in symptom domain scores postdialysis versus similar time points on nondialysis day.** MD <0=postdialysis symptom domain scores are higher. MD >0=postdialysis symptom domain scores are lower. For Negative Mood and Sleepiness/Fatigue, higher scores are worse. For Positive Mood and Alert Cognition, higher scores are better. MD, mean difference.

See Figures 1 and 2 for mean symptom domain scores at all postdialysis and nondialysis time points. In the postdialysis period, patients reported significantly lower Positive Mood (MD=−0.22; 95% confidence interval [CI], −0.29 to −0.14) and Alert Cognition (MD=−0.13; 95% CI, −0.18 to −0.08) and significantly higher Negative Mood (MD=0.12; 95% CI, 0.05 to 0.19) and Sleepiness/Fatigue symptoms (MD=0.51; 95% CI, 0.42 to 0.61) compared with similar time points on nondialysis days after adjusting for age, race, sex, and CCI. The sharpest increase in postdialysis symptoms was observed on the first hemodialysis day of the week (*i.e*., the day after having 2 consecutive days off of dialysis; Figures 1 and 2). These results did not change meaningfully when the second consecutive nondialysis day symptom domain scores were excluded in sensitivity analysis (differences in MD estimates ranged from 0.002 to 0.047; all *P* values remained < 0.001), nor did they meaningfully change after controlling for baseline Functional Assessment of Chronic Illness Therapy–Fatigue scores (see Supplemental Table 3). Figure 3 shows the MD in symptom domain scores during the postdialysis period versus the same period on nondialysis days.

The mean postdialysis symptom exacerbation total score was 0.70±1.21, and this represents an increase of 6% of the maximum scale range. In the postdialysis period, relative to nondialysis days, 58 patients (38%) endorsed a postdialysis increase in symptom severity of at least 1 point and 101 patients (64%) had at least 5% increase in their symptom exacerbation score. In the postdialysis period, relative to nondialysis days, the mean decrease in Positive Mood and Alert Cognition scores equaled 4% (95% CI, 2% to 5%) and 2% (95% CI, 1% to 3%) of the maximum scale range, respectively; the mean increase in Negative Mood and Sleepiness/Fatigue scores equaled 2% (95% CI, 1% to 3%) and 9% (95% CI, 7% to 10%) of the maximum scale range, respectively. Across all days, we observed 0.49 increase in Sleepiness/Fatigue scores from wake-up time to bedtime. Using this as a clinically meaningful anchor, the mean increase in Sleepiness/Fatigue scores in the postdialysis period, relative to nondialysis days, equaled 104%.

### Item-Level Analyses

After adjusting for age, race, sex, and CCI, when compared with their nondialysis days, patients reported on postdialysis days higher levels of stress, tension, irritability, fatigue, sleepiness, and exhaustion as well as significantly less happiness, energy, efficiency, alertness, and clear-headedness; they also endorsed greater difficulty concentrating and reported that it felt like everything took greater effort (*P* value range = < 0.001–0.01). No significant differences were observed for nondialysis versus postdialysis days on items reflecting feelings of being relaxed, calm, anxious, sad, or forgetful (*P* value range = 0.07–0.46). See Table 2 for adjusted analyses and Supplemental Table 4 for unadjusted analyses.

**Table 2:** Item-level analysis of postdialysis symptom exacerbation adjusted for age, sex, race, and Charlson Comorbidity Index

Item	Postdialysis Mean±SD	Nondialysis Mean±SD	Mean Difference (95% CI)
**Positive Mood**			
Relaxed	4.5±1.8	4.6±1.9	0.08 (−0.04 to 0.20)
Energetic	3.7±1.7	4.1±1.7	0.44^a^ (0.33 to 0.55)
Calm	4.7±1.8	4.8±1.8	0.11 (−0.01 to 0.22)
Happy	4.6±1.8	4.8±1.8	0.23^a^ (0.12 to 0.33)
Efficient	4.2±1.7	4.4±1.7	0.21^a^ (0.11 to 0.31)
**Negative Mood**			
Anxious	2.4±1.7	2.3±1.6	−0.07 (−0.17 to 0.03)
Stressed	2.7±1.8	2.5±1.7	−0.14^a^ (−0.25 to −0.04)
Tense	2.6±1.8	2.5±1.7	−0.17^a^ (−0.27 to −0.08)
Sad	2.2±1.6	2.1±1.7	−0.03 (−0.12 to 0.06)
Irritable	2.8±1.8	2.6±1.8	−0.20^a^ (−0.31 to −0.09)
**Alert Cognition**			
Forgetful^b^	5.3±1.7	5.4±1.7	0.05 (−0.05 to 0.16)
Clear-headed	4.8±1.8	5.2±1.7	0.34^a^ (0.23 to 0.45)
Concentrate	5.2±1.7	5.4±1.6	0.23^a^ (0.13 to 0.32)
Effort^b^	4.2±1.9	4.0±1.9	−0.25^a^ (−0.36 to −0.14)
Alert	4.9±1.9	5.2±1.8	0.29^a^ (0.17 to 0.40)
**Sleepiness/Fatigue**			
Fatigued	4.1±1.9	3.7±1.9	−0.42^a^ (−0.53 to −0.30)
Sleepy	4.1±2.0	3.5±1.9	−0.55^a^ (−0.68 to −0.43)
Exhausted	4.2±2.0	3.7±2.0	−0.57^a^ (−0.69 to −0.45)

Postdialysis=mean item scores for all time points after dialysis on dialysis days; nondialysis=mean item scores for the same time points on nondialysis days. For Positive Mood and Alert Cognition, lower scores are worse; for Negative Mood and Sleepiness/Fatigue, higher scores are worse. CI, confidence interval.

a Estimates were significant at the *P* < 0.05 level.

b Forgetful and effort items are reverse scored so that higher scores indicate patients are less forgetful and that it is less of an effort to do things.

### Predictors of Postdialysis Symptom Exacerbation

The results from the exploratory analysis of associations of sociodemographic, psychosocial, disease-specific, and hemodialysis treatment characteristics with postdialysis symptom exacerbation scores are presented in Table 3. After adjusting for age, race, sex, and CCI, the following characteristics were associated with greater postdialysis symptom exacerbation: higher hemoglobin (*β*=0.14; 95% CI, 0.04 to 0.24), *β* blocker medication use (*β*=0.39; 95% CI, 0.09 to 0.68), the first dialysis day after the long interdialytic gap (*β*=0.54; 95% CI, 0.21 to 0.86), lower baseline fatigue (*β*=0.02; 95% CI, 0.003 to 0.03), and higher social support (*β*=0.01; 95% CI, 0.003 to 0.02). The following characteristics were associated with lower postdialysis symptom exacerbation: White race (*β*=−0.43; 95% CI, −0.74 to −0.12), opioid medication use (*β*=−0.31; 95% CI, −0.61 to −0.01), and higher pain (*β*=−0.05; 95% CI, −0.09 to −0.01). See Supplemental Table 5 for unadjusted analyses.

**Table 3:** Associations of sociodemographic, disease-specific, treatment-related, and psychosocial characteristics with postdialysis symptom exacerbation adjusted for age, sex, race, and Charlson Comorbidity Index

Characteristic	Estimate (95% CI)
**Sociodemographic**	
Age, yr	0.01 (−0.0000004 to 0.02)
Sex, men^a^	0.20 (−0.08 to 0.47)
Race, White^b^	−0.43^c^ (−0.74 to −0.12)
Race, other^b^	−0.28 (−0.68 to 0.12)
Social Deprivation Index	0.001 (−0.004 to 0.01)
**Disease-specific**	
Charlson Comorbidity Index	−0.05 (−0.12 to 0.03)
Years on dialysis	0.02 (−0.01 to 0.05)
Hemoglobin, g/dl	0.14^c^ (0.04 to 0.24)
Albumin, g/dl	0.03 (−0.36 to 0.41)
Opioid medication use^d^	−0.31^c^ (−0.61 to −0.01)
Antidepressant medication use^d^	0.08 (−0.22 to 0.38)
Antihypertensive medication use^d^	0.03 (−0.35 to 0.40)
*β* blocker medication use^d^	0.39^c^ (0.09 to 0.68)
**Hemodialysis treatment**	
Dialysis schedule, Tuesday-Thursday-Saturday^e^	−0.24 (−0.53 to 0.04)
Dialysis shift time, early morning^f^	−0.07 (−1.66 to 1.52)
Dialysis shift time, midday^f^	−0.04 (−1.63 to 1.56)
Dialysis day, first^g^	0.54^c^ (0.21 to 0.86)
Dialysis day, second^g^	0.01 (−0.32 to 0.34)
Dialysis duration, min	−0.10 (−0.41 to 0.20)
Dialysis adequacy, kt/v	−0.07 (−0.46 to 0.32)
**Psychosocial**	
Functional Assessment of Chronic Illness Therapy–Fatigue	0.02^c^ (0.003 to 0.03)
Pittsburgh Sleep Quality Index	−0.01 (−0.05 to 0.03)
Brief Pain Inventory-short form	−0.05^c^ (−0.09 to −0.01)
Beck Depression Inventory II	−0.002 (−0.02 to 0.01)
General Anxiety Disorder-7	−0.02 (−0.05 to 0.01)
Physical Activity Scale for the Elderly	0.002 (−0.0000000004 to 0.01)
Multidimensional Scale of Perceived Social Support	0.01^c^ (0.003 to 0.02)

Postdialysis symptom exacerbation scores=the degree to which patients' Negative Mood and Sleepiness/Fatigue scores (summed) increase in the postdialysis period compared with their ratings on nondialysis days; higher scores indicate greater postdialysis symptom exacerbation. The “Other” race/ethnicity category includes all patients who identified as Hispanic, American Indian, or multiracial. CI, confidence interval.

a Estimates are based on women.

b Estimates are based on Black race.

c Estimates were significant at the *P* < 0.05 level.

d Estimates are based on no use at baseline.

e Estimates are based on Monday-Wednesday-Friday.

f Estimates are based on evening shift.

g Estimates are based on third dialysis day as reference groups.

## Discussion

To the best of our knowledge, this is the first study to use EMA to closely examine the physical, cognitive, and mood symptoms that patients report postdialysis. In this large, diverse sample of patients with kidney failure, we observed significant elevations in a range of symptoms during the postdialysis period compared with the same period on nondialysis days.

Our findings align with previous studies using retrospective self-report measures of fatigue, which have shown postdialysis fatigue to be highly prevalent among hemodialysis patients.^12^ The results from the item-level analyses are also closely aligned with findings from previous qualitative studies in which patients have described debilitating postdialysis fatigue, cognitive symptoms, and psychological symptoms that negatively affect their ability to complete tasks and fulfill role obligations after dialysis,^15^ and with quantitative studies showing these symptoms often cluster together with fatigue in those with kidney failure.^41,42^ However, it is important to note that although we found statistically significant differences indicating patients experience a greater burden of physical, cognitive, and mood symptoms in the postdialysis period, many of these differences were small. Furthermore, we are not able to draw definitive conclusions regarding the clinical significance of our findings as meaningful change thresholds have not been established for the DISS.

In our previous study,^10^ we reasoned that the increase in Sleepiness/Fatigue scores that patients report from wake-up to bedtime would provide a clinically meaningful anchor to which we could compare our findings. Although the modest 0.51 point increase in Sleepiness/Fatigue scores associated with postdialysis period in adjusted analyses is of uncertain clinical significance, it represents approximately 104% of the increase in Sleepiness/Fatigue scores associated with progression from wake-up to bedtime. In studies of the Insomnia Daytime Symptoms and Impacts Questionnaire (IDSIQ), a 14-item EMA measure adapted from the DISS, meaningful change thresholds have been identified for each subscale: 9 points for Alert Cognition scale (15% of the 60-point subscale range) and 4 points for the Mood and Sleepiness/Fatigue scales (10% of the 40-point subscale range).^43,44^ Applying this convention, the MDs we observed in postdialysis versus nondialysis DISS scores fall below these thresholds in our study (2%–9% for each domain), although the 95% CI around the MD in Sleepiness/Fatigue scores does include the 10% threshold, suggesting that this may be clinically significant. However, our findings may not be comparable with those of studies using the IDSIQ as there are notable differences in the measures used (*i.e*., half of the IDSIQ items were modified from the original DISS; respondents rate symptom severity on an 11-point scale for the IDSIQ versus a 7-point scale on the DISS), populations of interest (*i.e*., all IDSIQ studies included participants with insomnia and not kidney failure), and timing of measurement (within person change over 3 months versus diurnal change in ours).^43,44^

Although relatively little is known about the pathophysiology underlying postdialysis symptom exacerbation, it has been hypothesized that postdialysis fatigue could result from multiple etiologies.^31^ Although some previous studies have linked factors such as older age,^20–22^ greater dialysis vintage,^19,20^ and depressive symptoms^17,25–28^ to higher rates of postdialysis fatigue, these associations have not been consistently observed in other studies (see ref. 13 for a review). It is possible that heterogeneity in the instruments used to measure postdialysis fatigue could be contributing to these mixed findings, especially because little is known about the psychometric properties of postdialysis fatigue measures.^45^ For example, some postdialysis fatigue assessments could inadvertently measure the presence of fatigue rather than the degree to which fatigue worsens in the postdialysis period.^16^ Because EMA has been shown to reduce recall bias, our methods may have provided a more precise estimate of postdialysis symptom exacerbation.^34,36^ Our study provides hypothesis-generating exploratory data on underlying factors that may contribute to postdialysis symptom exacerbations. However, it is possible that other factors, not assessed in this study, could play a role (*e.g*., ultrafiltration rate, intradialytic hypotension, or hemodialysis prescription). For example, a recent study by Rose *et al.*^46^ showed that patients treated with high-dose hemodiafiltration had a significantly slower decline in health-related quality of life over time compared with those treated with hemodialysis. Considering high-dose hemodiafiltration may more effectively eliminate larger molecules (*e.g*., myoglobin, cytokines, and uremic “middle molecules”), future studies should examine whether the accumulation of these molecules could have a role in posthemodialysis symptom exacerbation.

Interestingly, patients tended to report the sharpest increase in postdialysis symptoms on their first hemodialysis day (*i.e*., the day after having 2 consecutive days off of dialysis). Previous studies have shown that hemodialysis patients have higher rates of emergency department visits, hospitalizations, and mortality on the day after the 2-day interdialytic gap compared with other hemodialysis days.^47–51^ It is plausible that increased fluid accumulation necessitating increased ultrafiltration rate, electrolyte disturbances, and increased “uremic toxins” after the 2-day interdialytic gap contribute to postdialysis symptom exacerbation.^52^ This is consistent with previous studies that have shown that interdialytic weight gain and skipped hemodialysis sessions are associated with greater symptom burden.^53,54^

Our study has many strengths, including the large sample size of patients who were diverse in terms of age, race, ethnicity, sex, comorbidity burden, and dialysis vintage. We also used an EMA approach to minimize recall bias in patient-reported symptoms with a high data completion rate. Limitations of this study include only assessing postdialysis symptoms at four time points during the day for 7 days. Including additional time points and a longer follow-up time could provide a more detailed understanding and help determine whether these patterns are consistent from week to week, but patient burden with completing more frequent assessments may limit this. Although we did assess several physical, cognitive, and mood states with the DISS, there are several important symptoms we did not examine such as nausea, vomiting, cramping, headaches, pain, dizziness, and loss of appetite. These symptoms have all been reported to occur or worsen during the postdialysis period^31,55^ and should be assessed in future EMA studies. Finally, owing to the inclusion criteria for the Technology-Assisted Collaborative Care trial,^38^ all patients in this study screened positive for either depression, pain, or fatigue before enrollment; although these symptoms are common in hemodialysis patients,^1,4,5,16^ our findings may not be generalizable to patients with a lower symptom burden.

Overall, our findings echo the remarks made by patients, clinicians, and researchers at the recent National Institute of Diabetes and Digestive and Kidney Diseases scientific workshop on postdialysis fatigue^31^ that many patients experience a wide range of symptoms beyond fatigue, including both negative mood and cognitive symptoms.^18,31^ In light of our findings, we agree with recent critiques that the term “postdialysis fatigue” fails to adequately characterize the complex, debilitating symptoms that patients experience after dialysis,^32,33^ as well as the calls for a new term.^18,31^ As such, we recommend use of the term “postdialysis syndrome (PDS)” to describe the acute exacerbation of physical, cognitive, and/or psychiatric symptoms that patients may experience after dialysis. Calvo *et al.*^56^ define a syndrome as “a recognizable complex of symptoms and physical findings which indicate a specific condition for which a direct cause is not necessarily understood.” We believe that this term accurately reflects the current state of the science on postdialysis symptom exacerbation and more accurately reflects the diversity of symptoms that patients may experience.^31–33^

Many patients with kidney failure on thrice-weekly maintenance in-center hemodialysis experience an increased burden of several physical, cognitive, and psychiatric symptoms in the period after their hemodialysis sessions. Retiring the term “postdialysis fatigue” and replacing it with “PDS” would more accurately describe this phenomenon and may help increase awareness of the complex symptom experiences that these patients face. More studies are needed to understand the etiology, establish a clear case definition, characterize related symptoms, and develop diagnostic tools and treatments for PDS.

## Supplementary Material

SUPPLEMENTARY MATERIAL

## Data Availability

Anonymized data created for the study are available in a persistent repository. Analyzable Data. GitHub. https://github.com/Renal-Electrolyte/TACcare-PDS-Public-Data-Sharing.

## Supplemental Material

Supplemental Table 1. Description of baseline measures of sample characteristics.

Supplemental Table 2. Sample characteristics by dialysis shift.

Supplemental Table 3. Symptom domain score analysis of postdialysis symptom exacerbation adjusted for age, sex, race, Charlson Comorbidity Index, and Functional Assessment of Chronic Illness Therapy–Fatigue score.

Supplemental Table 4. Unadjusted symptom domain score and item-level analysis of postdialysis symptom exacerbation.

Supplemental Table 5. Unadjusted associations of sociodemographic, disease-specific, and psychosocial characteristics with postdialysis symptom exacerbation.

## References

[1] Flythe JE Hilliard T Castillo G, . Symptom prioritization among adults receiving in-center hemodialysis: a mixed methods study. Clin J Am Soc Nephrol. 2018;13(5):735–745. doi:10.2215/CJN.10850917 29559445 PMC5969481

[2] Mehrotra R Davison SN Farrington K, . Managing the symptom burden associated with maintenance dialysis: conclusions from a Kidney Disease: Improving Global Outcomes (KDIGO) Controversies Conference. Kidney Int. 2023;104(3):441–454. doi:10.1016/j.kint.2023.05.019 37290600

[3] Fletcher BR Damery S Aiyegbusi OL, . Symptom burden and health-related quality of life in chronic kidney disease: a global systematic review and meta-analysis. PLoS Med. 2022;19(4):e1003954. doi:10.1371/journal.pmed.1003954 35385471 PMC8985967

[4] Murtagh FEM Addington-Hall J Higginson IJ. The prevalence of symptoms in end-stage renal disease: a systematic review. Adv Chronic Kidney Dis. 2007;14(1):82–99. doi:10.1053/j.ackd.2006.10.001 17200048

[5] Weisbord SD Fried LF Arnold RM, . Prevalence, severity, and importance of physical and emotional symptoms in chronic hemodialysis patients. J Am Soc Nephrol. 2005;16(8):2487–2494. doi:10.1681/ASN.2005020157 15975996

[6] Ng MSN Wong CL Ho EHS Hui YH Miaskowski C So WKW. Burden of living with multiple concurrent symptoms in patients with end-stage renal disease. J Clin Nurs. 2020;29(13-14):2589–2601. doi:10.1111/jocn.15282 32279368

[7] Claxton RN Blackhall L Weisbord SD Holley JL. Undertreatment of symptoms in patients on maintenance hemodialysis. J Pain Symptom Manage. 2010;39(2):211–218. doi:10.1016/j.jpainsymman.2009.07.003 19963337

[8] Caplin B Kumar S Davenport A. Patients’ perspective of haemodialysis-associated symptoms. Nephrol Dial Transplant. 2011;26(8):2656–2663. doi:10.1093/ndt/gfq763 21212166

[9] Parfrey PS Vavasour HM Henry S Bullock M Gault MH. Clinical features and severity of nonspecific symptoms in dialysis patients. Nephron. 1988;50(2):121–128. doi:10.1159/000185141 3065660

[10] Abdel-Kader K Jhamb M Mandich LA, . Ecological momentary assessment of fatigue, sleepiness, and exhaustion in ESKD. BMC Nephrol. 2014;15(1):29. doi:10.1186/1471-2369-15-29 24502751 PMC3927224

[11] Debnath S Rueda R Bansal S Kasinath BS Sharma K Lorenzo C. Fatigue characteristics on dialysis and non-dialysis days in patients with chronic kidney failure on maintenance hemodialysis. BMC Nephrol. 2021;22(1):112. doi:10.1186/s12882-021-02314-0 33773596 PMC7999524

[12] Dou J Liu H Ma Y Wu Y-Y Tao X-B. Prevalence of post-dialysis fatigue: a systematic review and meta-analysis. BMJ Open. 2023;13(6):e064174. doi:10.1136/bmjopen-2022-064174 PMC10277119 37311633

[13] Bossola M Hedayati SS Brys ADH Gregg LP. Fatigue in patients receiving maintenance hemodialysis: a review. Am J Kidney Dis. 2023;82(4):464–480. doi:10.1053/j.ajkd.2023.02.008 37187283 PMC11571972

[14] Sakkas GK Karatzaferi C. Hemodialysis fatigue: just “simple” fatigue or a syndrome on its own right? Front Physiol. 2012;3:306. doi:10.3389/fphys.2012.00306 22934057 PMC3429077

[15] Jacobson J Ju A Baumgart A, . Patient perspectives on the meaning and impact of fatigue in hemodialysis: a systematic review and thematic analysis of qualitative studies. Am J Kidney Dis. 2019;74(2):179–192. doi:10.1053/j.ajkd.2019.01.034 30955947

[16] Jhamb M Weisbord SD Steel JL Unruh M. Fatigue in patients receiving maintenance dialysis: a review of definitions, measures, and contributing factors. Am J Kidney Dis. 2008;52(2):353–365. doi:10.1053/j.ajkd.2008.05.005 18572290 PMC2582327

[17] Sklar AH Riesenberg LA Silber AK Ahmed W Ali A. Postdialysis fatigue. Am J Kidney Dis. 1996;28(5):732–736. doi:10.1016/s0272-6386(96)90256-5 9158212

[18] Reddy YNV O’Hare AM Edwards D Hall R. Defining, understanding, and addressing postdialysis fatigue: takeaways from a scientific workshop hosted by the National Institutes of Health. J Am Soc Nephrol. 2024;35(8):1130–1133. doi:10.1681/ASN.0000000000000409 39248633 PMC11377793

[19] Bossola M Di Stasio E Monteburini T, . Intensity, duration, and frequency of post-dialysis fatigue in patients on chronic haemodialysis. J Ren Care. 2020;46(2):115–123. doi:10.1111/jorc.12315 31984649

[20] Bossola M Marzetti E Di Stasio E, . Prevalence and associated variables of post-dialysis fatigue: results of a prospective multicentre study. Nephrology. 2018;23(6):552–558. doi:10.1111/nep.13059 28419668

[21] Bossola M Picca A Monteburini T, . Post-dialysis fatigue and survival in patients on chronic hemodialysis. J Nephrol. 2021;34(6):2163–2165. doi:10.1007/s40620-021-01141-8 34390478

[22] Han SJ Kim HW. Influencing Factors on Post-Dialysis Fatigue in Hemodialysis Patients. Healthcare and Nursing 2016; 2016:131–139. doi:10.14257/astl.2016.128.26.

[23] Bossola M Di Stasio E Monteburini T, . Recovery time after hemodialysis is inversely associated with the ultrafiltration rate. Blood Purif. 2019;47(1-3):45–51. doi:10.1159/000492919 30231240

[24] Bossola M Monteburini T Parodi E, . Post-dialysis fatigue: comparison of bicarbonate hemodialysis and online hemodiafiltration. Hemodial Int. 2023;27(1):55–61. doi:10.1111/hdi.13058 36404395

[25] Rayner HC Zepel L Fuller DS, . Recovery time, quality of life, and mortality in hemodialysis patients: the Dialysis Outcomes and Practice Patterns Study (DOPPS). Am J Kidney Dis. 2014;64(1):86–94. doi:10.1053/j.ajkd.2014.01.014 24529994 PMC4069238

[26] Gordon PL Doyle JW Johansen KL. Postdialysis fatigue is associated with sedentary behavior. Clin Nephrol. 2011;75(5):426–433. PMID: 21543022 21543022

[27] Lopes GB Silva LF Pinto GB, . Patient’s response to a simple question on recovery after hemodialysis session strongly associated with scores of comprehensive tools for quality of life and depression symptoms. Qual Life Res. 2014;23(8):2247–2256. doi:10.1007/s11136-014-0666-z 24627088

[28] Davenport A Guirguis A Almond M, . Postdialysis recovery time is extended in patients with greater self-reported depression screening questionnaire scores. Hemodial Int. 2018;22(3):369–376. doi:10.1111/hdi.12642 29461016

[29] Lindsay RM Heidenheim PA Nesrallah G Garg AX Suri R.; Daily Hemodialysis Study Group London Health Sciences Centre. Minutes to recovery after a hemodialysis session: a simple health-related quality of life question that is reliable, valid, and sensitive to change. Clin J Am Soc Nephrol. 2006;1(5):952–959. doi:10.2215/CJN.00040106 17699312

[30] Dreisbach AW Hendrickson T Beezhold D Riesenberg LA Sklar AH. Elevated levels of tumor necrosis factor alpha in postdialysis fatigue. Int J Artif Organs. 1998;21(2):83–86. doi:10.1177/039139889802100204 9569129

[31] National Institute of Diabetes and Digestive and Kidney Diseases. A Scientific Workshop on Post-Dialysis Fatigue - 2023; 2023. Accessed January 5, 2024. https://www.niddk.nih.gov/news/meetings-workshops/2023/scientific-workshop-on-post-dialysis-fatigue

[32] Chauhan K Wen HH Gupta N Nadkarni G Coca S Chan L. Higher symptom frequency and severity after the long interdialytic interval in patients on maintenance intermittent hemodialysis. Kidney Int Rep. 2022;7(12):2630–2638. doi:10.1016/j.ekir.2022.09.032 36506245 PMC9727533

[33] Zhou M Gu X Cheng K Wang Y Zhang N. Exploration of symptom clusters during hemodialysis and symptom network analysis of older maintenance hemodialysis patients: a cross-sectional study. BMC Nephrol. 2023;24(1):115. doi:10.1186/s12882-023-03176-4 37106315 PMC10132956

[34] Shiffman S Stone AA Hufford MR. Ecological momentary assessment. Annu Rev Clin Psychol. 2008;4:1–32. doi:10.1146/annurev.clinpsy.3.022806.091415 18509902

[35] Schneider SM Malecki AK Müller K, . Effect of a single dialysis session on cognitive function in CKD5D patients: a prospective clinical study. Nephrol Dial Transplant. 2015;30(9):1551–1559. doi:10.1093/ndt/gfv213 26071228

[36] Brys ADH Stifft F Van Heugten CM Bossola M Gambaro G Lenaert B. Unraveling fatigue in hemodialysis patients: comparing retrospective reports to real-time assessments with an mHealth experienced sampling method. J Pain Symptom Manage. 2020;60(6):1100–1108.e2. doi:10.1016/j.jpainsymman.2020.06.042 32645453

[37] Riis J Loewenstein G Baron J Jepson C Fagerlin A Ubel PA. Ignorance of hedonic adaptation to hemodialysis: a study using ecological momentary assessment. J Exp Psychol Gen. 2005;134(1):3–9. doi:10.1037/0096-3445.134.1.3 15702959

[38] Jhamb M Steel JL Yabes JG, . Effects of technology assisted stepped collaborative care intervention to improve symptoms in patients undergoing hemodialysis: the TĀCcare randomized clinical trial. JAMA Intern Med. 2023;183(8):795–805. doi:10.1001/jamainternmed.2023.2215 37338898 PMC10282960

[39] Roumelioti ME Steel JL Yabes J, . Rationale and design of technology assisted stepped collaborative care intervention to improve patient-centered outcomes in hemodialysis patients (TĀCcare trial). Contemp Clin Trials. 2018;73(6):81–91. doi:10.1016/j.cct.2018.09.002 30208343 PMC6168366

[40] Buysse DJ Thompson W Scott J, . Daytime symptoms in primary insomnia: a prospective analysis using ecological momentary assessment. Sleep Med. 2007;8(3):198–208. doi:10.1016/j.sleep.2006.10.006 17368098 PMC1899354

[41] Jhamb M Abdel-Kader K Yabes J, . Comparison of fatigue, pain, and depression in patients with advanced kidney disease and cancer—symptom burden and clusters. J Pain Symptom Manage. 2019;57(3):566–575.e3. doi:10.1016/j.jpainsymman.2018.12.006 30552961 PMC6382584

[42] Cao X Tian L Lin C. Symptom clusters in patients receiving haemodialysis: a systematic review of observational studies. J Clin Nurs. 2017;26(17-18):2545–2557. doi:10.1111/jocn.13644 27862490

[43] Hudgens S Phillips-Beyer A Newton L Seboek Kinter D Benes H. Development and validation of the Insomnia Daytime Symptoms and Impacts Questionnaire (IDSIQ). Patient. 2021;14(2):249–268. doi:10.1007/s40271-020-00474-z 33131027 PMC7884372

[44] Phillips-Beyer A Kawata AK Kleinman L Kinter DS. Meaningful within-patient change on the Insomnia Daytime Symptoms and Impacts Questionnaire (IDSIQ): analysis of phase III clinical trial data of daridorexant. Pharm Med. 2023;37(4):291–303. doi:10.1007/s40290-023-00484-w PMC10314845 37286927

[45] Ju A Unruh ML Davison SN, . Patient-reported outcome measures for fatigue in patients on hemodialysis: a systematic review. Am J Kidney Dis. 2018;71(3):327–343. doi:10.1053/j.ajkd.2017.08.019 29198388

[46] Rose M Fischer FH Liegl G, . The CONVINCE randomized trial found positive effects on quality of life for patients with chronic kidney disease treated with hemodiafiltration. Kidney Int. 2024;106(5):961–971. doi:10.1016/j.kint.2024.07.014 39089577

[47] Foley RN Gilbertson DT Murray T Collins AJ. Long interdialytic interval and mortality among patients receiving hemodialysis. New Engl J Med. 2011;365(12):1099–1107. doi:10.1056/NEJMoa1103313 21992122

[48] Zhang H Schaubel DE Kalbfleisch JD, . Dialysis outcomes and analysis of practice patterns suggests the dialysis schedule affects day-of-week mortality. Kidney Int. 2012;81(11):1108–1115. doi:10.1038/ki.2011.481 22297673 PMC3365564

[49] Fotheringham J Fogarty DG El Nahas M Campbell MJ Farrington K. The mortality and hospitalization rates associated with the long interdialytic gap in thrice-weekly hemodialysis patients. Kidney Int. 2015;88(3):569–575. doi:10.1038/ki.2015.141 25970155

[50] Bleyer AJ Russell GB Satko SG. Sudden and cardiac death rates in hemodialysis patients. Kidney Int. 1999;55(4):1553–1559. doi:10.1046/j.1523-1755.1999.00391.x 10201022

[51] Zhang S Morgenstern H Albertus P Nallamothu BK He K Saran R. Emergency department visits and hospitalizations among hemodialysis patients by day of the week and dialysis schedule in the United States. PLoS One. 2019;14(8):e0220966. doi:10.1371/journal.pone.0220966 31415609 PMC6695146

[52] Rhee CM Kalantar-Zadeh K. Implications of the long interdialytic gap: a problem of excess accumulation vs. excess removal? Kidney Int. 2015;88(3):442–444. doi:10.1038/ki.2015.193 26323071 PMC4566144

[53] Alvarez L Brown D Hu D Chertow GM Vassalotti JA Prichard S. Intradialytic symptoms and recovery time in patients on thrice-weekly in-center hemodialysis: a cross-sectional online survey. Kidney Med. 2020;2(2):125–130. doi:10.1016/j.xkme.2019.10.010 32734233 PMC7380355

[54] Li H Yin J Dong Y Tian Z. Factors predicting post-dialysis fatigue of maintenance hemodialysis patients. Ren Replace Ther. 2023;9(1):30. doi:10.1186/s41100-023-00486-z

[55] Kodama H Togari T Konno Y, . A new assessment scale for post-dialysis fatigue in hemodialysis patients. Ren Replace Ther. 2020;6(1):1. doi:10.1186/s41100-019-0252-5

[56] Calvo F Karras BT Phillips R Kimball AM Wolf F. Diagnoses, syndromes, and diseases: a knowledge representation problem. AMIA Annu Symp Proc. 2003;2003:802. PMID: 14728307 14728307 PMC1480257

