# Supplementary material for: Using Ecological Momentary Assessment to Redefine Postdialysis Fatigue in Patients with Kidney Failure
Source: J Am Soc Nephrol. 2025 Feb 25;36(8):1603–13. doi: 10.1681/ASN.0000000650 (PMC12342076; doi:10.1681/ASN.0000000650)
Supplement: SUPPLEMENTARY MATERIAL [file jasn-36-1603-s002.pdf]

Supplemental Material

Using Ecological Momentary Assessment to Redefine Postdialysis Fatigue in Patients with Kidney Failure

Cramer J. Kallem, PsyD

Alaa A. Alghwiri, PhD

Jonathan G. Yabes, PhD

Sarah Erickson, PhD

Zhuoheng Han, MSP

Maria-Eleni Roumelioti, MD

Jennifer L. Steel, PhD

Mark Unruh, MD, MS

Manisha Jhamb, MD, MPH

Table of Contents:

Supplemental Table 1. Description of Baseline Measures of Sample Characteristics (pg. 2)

Supplemental Table 2. Sample Characteristics by Dialysis Shift (pg. 3)

Supplemental Table 3. Symptom Domain Score Analysis of Post-dialysis Symptom Exacerbation Adjusted for Age, Gender, Race, Charlson Comorbidity Index, and Functional Assessment of Chronic Illness Therapy-Fatigue Score

Supplemental Table 4. Unadjusted Symptom Domain Score and Item-Level Analysis of Post-dialysis Symptom Exacerbation (pg.4)

Supplemental Table 5. Unadjusted Associations of Sociodemographic, Disease-Specific, and Psychosocial Characteristics with Post-Dialysis Symptom Exacerbation (pg. 6)

Supplemental Table 1. Description of Baseline Measures of Sample Characteristics

Characteristic	Measure Name	Measure Description
**Sociodemographic**		
Age	Sociodemographic questionnaire*	Patient age in years
Gender	Sociodemographic questionnaire*	Patient gender (male, female)
Race	Sociodemographic questionnaire*	Patient race (American Indian, Asian, Black, White)
Ethnicity	Sociodemographic questionnaire*	Patient ethnicity (Hispanic or Latino, not Hispanic or Latino)
Social Determinants of Health Burden	Social Deprivation Index (SDI)*	Patient zip codes were used to calculate SDI scores. The SDI is a validated measure of area-level deprivation that has been associated with health outcomes. Scores range from 0 to 100 with greater scores indicating greater social disadvantage.[1]
**Disease-Specific**		
Comorbidity Burden	Charlson Comorbidity Index (CCI)†	The CCI is a 19-item measure of risk related to the presence and severity of various comorbid medical conditions; CCI scores range from 0 to 37 with higher scores indicating greater comorbidity burden and risk for poor health outcomes.[2]
Dialysis Vintage	Clinical Questionnaire*	Time since initiating dialysis in years
Hemoglobin	––†	Blood hemoglobin levels in g/dL
Albumin	––†	Blood albumin levels in g/dL
Opioid Use	Clinical Questionnaire*	Opioid prescription at baseline (yes, no)
Antidepressant Use	Clinical Questionnaire*	Antidepressant prescription at baseline (yes, no)
**Hemodialysis Treatment**		
Dialysis Shift	––†	Early morning, midday, or evening dialysis shift
Dialysis Schedule	––†	Weekly dialysis treatment schedule (Mon-Wed-Fri, Tue-Thu,Sat)
Dialysis Day	––†	First, second, or third dialysis treatment after the long interdialytic interval
Dialysis Duration	––†	Mean duration of dialysis sessions in hours
Dialysis Adequacy	––†	Adequacy of Dialysis in Kt/V
**Psychosocial**		
Fatigue	Functional Assessment of Chronic Illness Therapy-Fatigue (FACIT-F)*	The FACIT-F is a 13-item measure of fatigue and its effects on daily functioning; Total FACIT-F scores range from 0 to 53 with lower scores indicating greater fatigue.[3]
Sleep Quality	Pittsburgh Sleep Quality Index (PSQI)*	The PSQI is a 19-item measure of sleep quality; PSQI global sleep scores range from 0 to 21 with higher scores indicating poorer sleep.[4]
Pain	Brief Pain Inventory-Short Form (BPI-SF)*	The BPI-SF is a 9-item measure of pain intensity and interference; the BPI-SF "average pain intensity" item was used for this study and scores on this item range from 0 to 10 with higher scores indicating greater pain intensity.[5]
Depressive Symptoms	Beck Depression Inventory II (BDI-II)*	The BDI-II is a 21-item measure of depressive symptoms; Total BDI-II scores range from 0 to 63 with higher scores indicating greater depressive symptoms.[6]
Anxiety	General Anxiety Disorder-7 (GAD-7)*	The GAD-7 is a 7-item measure of anxiety symptoms; Total GAD-7 scores range from 0 to 21 with higher scores indicating greater anxiety.[7]
Physical Activity	Physical Activity Scale for the Elderly (PASE)*	The PASE is a 12-item measure of physical activity; Total PASE scores range from 0 to 400 with greater scores indicating greater physical activity.[8]
Social Support	Multidimensional Scale of Perceived Social Support (MSPSS)*	The MSPSS is a 12-item measure of one's perceived level of social support. Scores range from 12 to 84 with higher scores indicating greater perceived social support.[9]

Note: Method of data collection = *Patient self-report, †medical record review

Supplemental Table 2. Sample Characteristics by Dialysis Shift

Characteristic	Category or Statistic	Morning (n=75)	Midday or Evening (n=81)	Test of Difference (p-value)
**Sociodemographic**				
Age	Mean years ± SD	58.0 ± 12.8	57.4 ± 14.8	0.9
Gender	Female, n (%)	25 (33)	45 (56)	0.01
	Male, n (%)	50 (67)	36 (44)	-
Race	Black, n (%)	22 (29)	23 (28)	> 0.9
	White, n (%)	37 (49)	42 (52)	-
	Other, n (%)	16 (21)	16 (20)	-
Ethnicity	Hispanic, n (%)	11 (15)	15 (19)	0.5
	Non-Hispanic, n (%)	64 (85)	66 (81)	-
Social Deprivation Index	Mean ± SD	57.8 ± 31.4	52.9 ± 30.1	0.3
**Disease-Specific**				
CCI	Mean ± SD	4.6 ± 1.8	4.9 ± 1.8	0.2
Dialysis Vintage	Mean years ± SD	4.5 ± 4.4	3.8 ± 4.1	0.2
Hemoglobin	Mean g/dL ± SD	11.2 ± 1.2	11.1 ± 1.4	0.5
Albumin	Mean g/dL ± SD	4.0 ± 0.4	4.0 ± 0.4	0.8
Opioid Use	Yes, n (%)	18 (24)	28 (35)	0.13
Antidepressant Use	Yes, n (%)	19 (25)	38 (48)	0.004
Antihypertensive Use	Yes, n (%)	66 (88)	65 (81)	0.2
Beta Blocker Use	Yes, n (%)	51 (68)	54 (68)	>0.99
**Hemodialysis Treatment**				
Dialysis Schedule	Mon-Wed-Fri n (%)	51 (68)	49 (60)	0.3
	Tue-Thu-Sat, n (%)	24 (32)	32 (40)	-
Dialysis Duration	Mean hours ± SD	4.1 ± 0.4	4.0 ± 0.6	0.7
Dialysis Adequacy	Mean Kt/V ± SD	1.6 ± 0.3	1.6 ± 0.4	0.7
**Psychosocial**				
FACIT-F	Mean ± SD	30.2 ± 10.2	26.6 ± 11.5	0.02
PSQI	Mean ± SD	8.8 ± 3.1	9.0 ± 3.7	0.8
BPI-SF	Mean ± SD	3.7 ± 3.3	3.5 ± 3.3	0.6
BDI-II	Mean ± SD	13.4 ± 7.6	17.1 ± 8.7	0.01
GAD-7	Mean ± SD	4.9 ± 4.5	6.9 (4.8)	0.01
PASE	Mean ± SD	229.1 ± 49.6	229.2 ± 58.8	0.6
MSPSS	Mean ± SD	64.6 ± 12.2	61.1 ± 15.4	0.4
**Ecological Momentary Assessment**				
Non-Dialysis Symptom Domain Scores	Positive Mood, mean ± SD	4.6 ± 1.4	4.4 ± 1.3	0.13
	Negative Mood, mean ± SD	2.3 ± 1.3	2.6 ± 1.4	0.09
	Alert Cognition, mean ± SD	5.1 ± 0.9	5.0 ± 1.0	0.57
	Sleepiness/Fatigue, mean ± SD	4 ± 1.6	4.3 ± 1.8	0.11
Post-Dialysis Symptom Domain Scores	Positive Mood, mean ± SD	4.4 ± 1.3	4.2 ± 1.3	0.09
	Negative Mood, mean ± SD	2.4 ± 1.3	2.7 ± 1.5	0.09
	Alert Cognition, mean ± SD	4.9 ± 1.0	4.9 ± 0.9	0.98
	Sleepiness/Fatigue, mean ± SD	4.0 ± 1.6	4.3 ± 1.8	0.11

Abbreviations: Functional Assessment of Chronic Illness Therapy-Fatigue (FACIT-F); Pittsburgh Sleep Quality Index (PSQI); Brief Pain Inventory-Short Form (BPI-SF); Beck Depression Inventory II (BDI-II); General Anxiety Disorder-7 (GAD-7); Physical Activity Scale for the Elderly (PASE); Multidimensional Scale of Perceived Social Support (MSPSS).

Supplemental Table 3. Symptom Domain Score Analysis of Post-dialysis Symptom Exacerbation Adjusted for Age, Gender, Race, Charlson Comorbidity Index, and Functional Assessment of Chronic Illness Therapy-Fatigue Score

Symptom Domain	Mean Difference MD [95% CI]
Negative Mood	**-0.12** [-0.19,- 0.05]
Sleepiness/Fatigue	**-0.51** [-0.61, -0.42]
Positive Mood	**0.22** [0.14, 0.29]
Alert Cognition	**0.13** [0.08, 0.18]

Note: For Positive Mood and Alert Cognition, lower scores are worse, implying symptoms are worse on dialysis day; for Negative Mood and Sleepiness/Fatigue, higher scores are worse, implying symptoms are worse on dialysis day. Bolded estimates were significant at the $p<0.05$ level.

Supplemental Table 4. Unadjusted Symptom Domain Score and Item-Level Analyses of Post-dialysis Symptom Exacerbation

Symptom Domain or Item	Mean Difference MD [95% CI]
**Positive Mood**	**0.19** [0.09, 0.30]
Relaxed	0.04 [-0.10, 0.19]
Energetic	**0.41** [0.27, 0.55]
Calm	0.08 [-0.06, 0.22]
Happy	**0.22** [0.08, 0.36]
Efficient	**0.21** [0.08, 0.35]
**Negative Mood**	**-0.14** [-0.25, -0.03]
Anxious	-0.09 [-0.22, 0.05]
Stressed	**-0.15** [-0.29, -0.01]
Tense	**-0.19** [-0.32, -0.05]
Sad	-0.05 [-0.19, 0.08]
Irritable	**-0.22** [-0.36, -0.08]
**Alert Cognition**	**0.13** [0.05, 0.20]
Forgetful*	0.06 [-0.08, 0.20]
Clear-headed	**0.33** [0.19, 0.47]
Concentrate	**0.21** [0.08, 0.34]
Effort*	**-0.23** [-0.38, -0.08]
Alert	**0.27** [0.12, 0.42]
**Sleepiness/Fatigue**	**-0.49** [-0.62, -0.36]
Fatigued	**-0.39** [-0.54, -0.24]
Sleepy	**-0.54** [-0.69, -0.38]
Exhausted	**-0.55** [-0.70, -0.39]

Note: *Forgetful and *Effort items are reverse scored so that higher scores indicate patients are less forgetful and that it is less of an effort to do things. For Positive Mood and Alert Cognition, lower scores are worse; for Negative Mood and Sleepiness/Fatigue, higher scores are worse. Bolded estimates were significant at the $p<0.05$ level.

Supplemental Table 5. Unadjusted Associations of Sociodemographic, Disease-Specific, Treatment-related and Psychosocial Characteristics with Post-Dialysis Symptom Exacerbation

Characteristic	Estimate [95% CI]
**Sociodemographic**	
Age (years)	0.01 [-0.003, 0.02]
[*]Gender, Men	0.11 [-0.16, 0.38]
[†]Race, White	**-0.39** [-0.70, -0.08]
[†]Race, Other	-0.30 [-0.69, 0.10]
Social Deprivation Index	0.003 [-0.002, 0.01]
**Disease-Specific**	
Charlson Comorbidity Index	-0.02 [-0.09, 0.05]
Years on Dialysis	0.02 [-0.01, 0.05]
Hemoglobin (g/dL)	**0.14** [0.04, 0.24]
Albumin (g/dL)	0.07 [-0.31, 0.45]
[‡]Opioid Medication Use	**-0.32** [-0.62, -0.02]
[‡]Antidepressant Medication Use	-0.07 [-0.35, 0.22]
[‡]Antihypertensive Medication Use	0.01 [-0.36, 0.38]
[‡]Beta Blocker Medication Use	**0.36** [0.08, 0.65]
**Hemodialysis Treatment**	
[§]Dialysis Schedule, Tuesday-Thursday-Saturday	**-0.30** [-0.58, -0.02]
[¶]Dialysis Shift Time, Early Morning	0.17 [-1.40, 1.75]
[¶]Dialysis Shift Time, Midday	0.16 [-1.42, 1.73]
[#]Dialysis Day, First	**0.53** [0.21, 0.86]
[#]Dialysis Day, Second	0.01 [-0.32, 0.33]
Dialysis Duration (minutes)	-0.04 [-0.32, 0.25]
Dialysis Adequacy (kt/v)	-0.11 [-0.49, 0.26]
**Psychosocial**	
Functional Assessment of Chronic Illness Therapy-Fatigue	**0.02** [0.004, 0.03]
Pittsburgh Sleep Quality Index	-0.02 [-0.06, 0.02]
Brief Pain Inventory-Short Form	-0.04 [-0.08, 0.00]
Beck Depression Inventory II	-0.01 [-0.02, 0.01]
General Anxiety Disorder-7	**-0.03** [-0.06, -0.002]
Physical Activity Scale for the Elderly	0.001 [-0.002, 0.003]
Multidimensional Scale of Perceived Social Support	**0.01** [0.002, 0.02]

Note: Post-dialysis symptom exacerbation scores = the degree to which patients' Negative Mood and Sleepiness/Fatigue scores (summed) increase in the post-dialysis period compared to their ratings on non-dialysis days; higher scores indicate greater post-dialysis symptom exacerbation. Estimates are based on [*]women, [†]Black, [‡]no use at baseline, [§]Monday-Wednesday-Friday, [¶]evening shift, and [#]third dialysis day as reference groups. The "Other" race/ethnicity category includes all patients who identified as Hispanic, American Indian, or multiracial. Bolded estimates were significant at the $p<0.05$ level.

References for Supplemental Table 1

1. Butler DC, Petterson S, Phillips RL, Bazemore AW. Measures of social deprivation that predict health care access and need within a rational area of primary care service delivery. *Health Serv Res*. 2013;48(2 Pt 1):539-559. doi:10.1111/j.1475-6773.2012.01449.x

2. Charlson ME, Carrozzino D, Guidi J, Patierno C. Charlson Comorbidity Index: A Critical Review of Clinimetric Properties. *Psychother Psychosom*. 2022;91(1):8-35. doi:10.1159/000521288

3. Webster K, Cella D, Yost K. The Functional Assessment of Chronic Illness Therapy (FACIT) Measurement System: properties, applications, and interpretation. *Health Qual Life Outcomes*. 2003;1:79. doi:10.1186/1477-7525-1-79

4. Buysse DJ, Reynolds CF, Monk TH, Berman SR, Kupfer DJ. The Pittsburgh sleep quality index: A new instrument for psychiatric practice and research. *Psychiatry Research*. 1989;28(2):193-213. doi:10.1016/0165-1781(89)90047-4

5. Keller S, Bann CM, Dodd SL, Schein J, Mendoza TR, Cleeland CS. Validity of the Brief Pain Inventory for Use in Documenting the Outcomes of Patients With Noncancer Pain. *The Clinical Journal of Pain*. 2004;20(5):309.

6. Wang YP, Gorenstein C. Psychometric properties of the Beck Depression Inventory-II: a comprehensive review. *Braz J Psychiatry*. 2013;35(4):416-431. doi:10.1590/1516-4446-2012-1048

7. Williams N. The GAD-7 questionnaire. *Occupational Medicine*. 2014;64(3):224. doi:10.1093/occmed/kqt161

8. Washburn RA, Smith KW, Jette AM, Janney CA. The Physical Activity Scale for the Elderly (PASE): development and evaluation. *J Clin Epidemiol*. 1993;46(2):153-162. doi:10.1016/0895-4356(93)90053-4

9. Kazarian SS, McCabe SB. Dimensions of social support in the MSPSS: Factorial structure, reliability, and theoretical implications. *Journal of Community Psychology*. 1991;19(2):150-160. doi:10.1002/1520-6629(199104)19:2<150::AID-JCOP2290190206>3.0.CO;2-J
